# Coding-Complete Genome Sequences of NITMA1086 and NITMA1139, Two SARS-CoV-2 Isolates from Belagavi District, Karnataka State, India, Harboring the D614G Mutation

**DOI:** 10.1128/MRA.00016-21

**Published:** 2021-02-11

**Authors:** Ishwar Singh, Umashankar Vetrivel, D. R. Harish, Debprasad Chattophadhyay

**Affiliations:** a National Institute of Traditional Medicine, Indian Council of Medical Research (ICMR), Department of Health Research (Government of India), Nehru Nagar, Belagavi, Karnataka, India; Queens College

## Abstract

We announce the coding-complete genome sequences of two isolates of severe acute respiratory syndrome coronavirus 2 (SARS-CoV-2) from two coronavirus disease 2019 (COVID-19)-positive samples (RNA isolated from nasopharyngeal swabs) from Belagavi District, Karnataka State, India. Mutational analysis revealed the presence of the D614G substitution in both the isolates.

## ANNOUNCEMENT

Severe acute respiratory syndrome coronavirus 2 (SARS-CoV-2) infection was first reported in China during December 2019 (1). The virus belongs to the genus *Betacoronavirus* under the family *Coronaviridae*. The first case of coronavirus disease 2019 (COVID-19) in India was reported on 30 January 2020 (2). Currently, India has the second highest number of confirmed cases, after the United States. Hence, it is important to understand the spread of virus strains, from district to country level. Here, we report the coding-complete genome sequences of two SARS-CoV-2 isolates, NITMA1086 and NITMA1139, from Belagavi District, Karnataka State, India. First, RNA was extracted from nasopharyngeal swabs (using a Qiagen RNA extraction minikit) from two randomly selected reverse transcriptase PCR (RT-PCR)-positive (DiAGSure nCoV-19 detection assay; GCC Biotech, India) samples.

The libraries for total/viral RNA sequencing were prepared based on the NEBNext RNA Ultra II directional protocol. The whole genomes were sequenced using the HiSeq X (Illumina, USA)-based paired-end (2 × 150-bp) sequencing protocol, which resulted in 12.41 Gb of data with 82 million reads for NITMA1086 and 2.97 Gb of data with 24.6 million reads for NITMA1139. The reads were subjected to adapter trimming using fastq-mcf v1.04.803 (https://github.com/ExpressionAnalysis/ea-utils/blob/wiki/FastqMcf.md). Subsequently, the trimmed reads were aligned to the human genome (hg19) sequence using BWA v0.7.12 (https://github.com/lh3/bwa/releases/tag/0.7.12) to remove the host sequences. This resulted in 28,488,328 host unaligned reads for NITMA1086 and 8,159,678 reads for NITMA1139. These unaligned reads were subjected to *de novo* assembly using the MetaSPAdes v3.11.1 assembler (3). This assembly resulted in 85,634 scaffolds for NITMA1086 (*N*_50_, 1,877 bp) and 52,335 scaffolds for NITMA1139 (*N*_50_, 1,035 bp). The assembled scaffolds were oriented to the Wuhan reference strain (GenBank accession number NC_045512.2) using RagTag (https://github.com/malonge/RagTag) (4) analysis, and the whole-genome sequences were retrieved. Subsequently, SNP-Sites (5) was used to perform variant calling of the whole-genome sequences with reference to the Wuhan strain (NC_045512.2). All bioinformatics software used in this study was run with default parameters. NITMA1086 had a sequence length of 29,849 bp (GC content, 38%), with an average sequence depth of 110.57× and 99.81% genome coverage. NITMA1139 had a sequence length of 29,854 bp (GC content, 38%), with an average sequence depth of 3,587.38× and 99.83% genome coverage.

Mutational analysis of NITMA1086 revealed 15 mutations, of which 8 were found to cause amino acid substitutions. In the case of NITMA1139, of the 16 mutations identified, 8 were found to cause substitutions. In both of the isolates, the P314L (ORF1b), D614G (S), R203K (N), G50N (ORF14), and G204R (N) substitutions were found in common (Table 1). Of these variations, D614G is reported to be highly prevalent worldwide and is associated with higher viral load and titers of pseudoviruses (6).

**TABLE 1:** List of mutations and amino acid changes observed in the sequenced genomes

Isolate name	Nucleotide change(s)/SNP^*a*^^,^^*b*^	Gene(s)	Variance/amino acid change(s)^*a*^
NITMA1086	*C241T*	Intergenic	*upstream_gene_variant*
NITMA1086	C2695T	ORF1ab	synonymous_variant
NITMA1086	*C3037T*	ORF1ab	*synonymous_variant*
NITMA1086	G8371T	ORF1a	Q2702H
NITMA1086	**C14408T**	ORF1b	**P314L**
NITMA1086	C18877T	ORF1ab	synonymous_variant
NITMA1086	G21468T	ORF1b	M2667I
NITMA1086	**A23403G**	S	**D614G**
NITMA1086	A24774T	S	Q1071L
NITMA1086	C24784T	S	synonymous_variant
NITMA1086	C26010T	ORF3a	synonymous_variant
NITMA1086	A28055G	ORF8	synonymous_variant
NITMA1086	**G28881A, G28882A**	N	**R203K**
NITMA1086	**G28883C**	N, ORF14	**G204R, G50N**
NITMA1139	*C241T*	Intergenic	*upstream_gene_variant*
NITMA1139	C313T	ORF1ab	synonymous_variant
NITMA1139	A4372G	ORF1ab	synonymous_variant
NITMA1139	*C3037T*	ORF1ab	*synonymous_variant*
NITMA1139	A5608G	ORF1ab	synonymous_variant
NITMA1139	C5700A	ORF1ab	A1812D
NITMA1139	C9693T	ORF1ab	A3143V
NITMA1139	G9190T	ORF1ab	synonymous_variant
NITMA1139	**C14408T**	ORF1ab	**P314L**
NITMA1139	C16626T	ORF1ab	synonymous_variant
NITMA1139	A18253G	ORF1ab	M15967V
NITMA1139	C18555T	ORF1ab	synonymous_variant
NITMA1139	C23230T	S	synonymous_variant
NITMA1139	**A23403G**	S	**D614G**
NITMA1139	**G28881A, G28882A**	N	**R203K**
NITMA1139	**G28883C**	N, ORF14	**G204R, G50N**

a Bold indicates the mutations leading to amino acid substitutions commonly observed in NITMA1086 and NITMA1139; italic indicates the other synonymous/intergenic variants commonly observed in NITMA1086 and NITMA1139.

b SNP, single nucleotide polymorphism.

**FIG 1:**
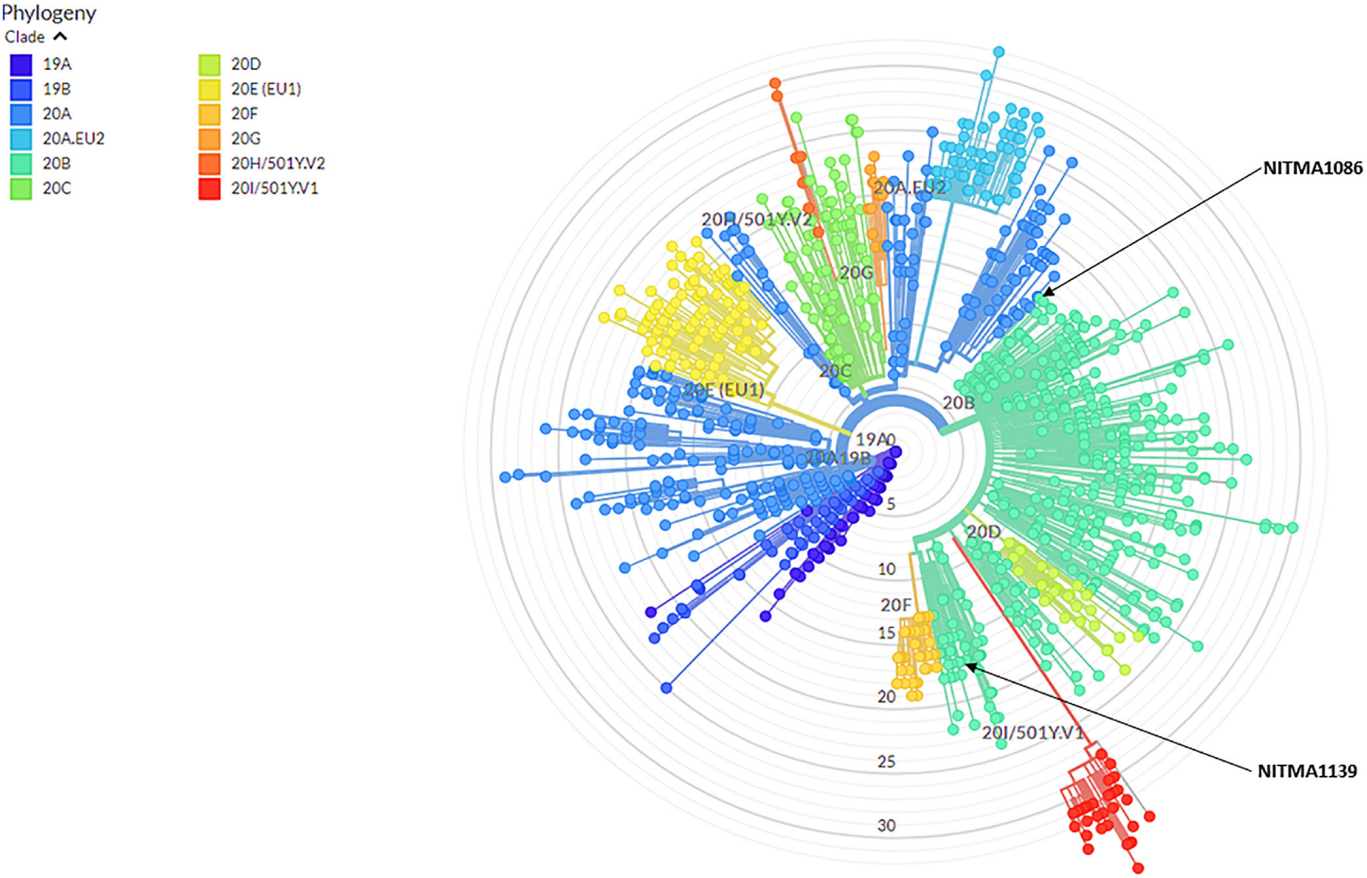
Phylogenetic tree displaying sequenced SARS-CoV-2 strains (with reference to GISAID global data set), in accordance to the clades assigned for the virus during phylogenetic analysis using the Augur toolkit (with default parameters) run through the Nextstrain server. Augur implements the FastTree algorithm, which infers the maximum-likelihood method for building phylogenetic trees from alignments of nucleotide or protein sequences (7, 10). Both of the sequenced strains are colored according to the assigned clade, complying with the recently established nomenclature (11). The locations of the two isolates, NITMA1089 and NITMA1139, are marked with black arrows.

Phylogenetic comparison of these two genome sequences with globally detected viral strains was performed using the Nextstrain tool (with an inbuilt global data set from GISAID) (7). This analysis revealed that both isolates belong to clade 20B, a subclade of 20A, which emerged in the European outbreak and evolved from the 19A Wuhan strain (Fig. 1). In the current scenario, 20B has been reported to be more common in the Indian population and is considered a major basis of disease transmission in India (8). Common sharing and collating of whole-genome sequences during viral outbreaks are essential as a crucial part of outbreak response (9). Thus, the genome sequences of these isolates will aid in studying the evolution and epidemiology of the virus and its transmission dynamics in Belagavi, as well as throughout India.

### Data availability.

These genome sequences have been deposited in the NCBI GenBank database under the accession numbers MW425563.1 and MW425837.1 for NITMA1086 and NITMA1139, respectively. The raw reads were also submitted to the NCBI SRA under the accession numbers SRX9766878 and SRX9766838 for NITMA1086 and NITMA1139, respectively.
